# Experience and Discussion: Safeguards for People With Disabilities During the COVID-19 Pandemic in China

**DOI:** 10.3389/fpubh.2021.744706

**Published:** 2021-11-04

**Authors:** Fei Qi, Yuqi Wu, Qi Wang

**Affiliations:** ^1^Law School, Hainan University, Haikou, China; ^2^Danzhou City Construction Investment Co., Ltd., Danzhou, China

**Keywords:** COVID-19 pandemic, disability empowerment, healthcare discrimination, people with disability, interactive decision-making system, vaccine distribution

## Abstract

The special vulnerability of people with disability (PWD) in the COIVD-19 pandemic has been confirmed by many studies, but data shows that the infection rate of PWD in China is lower than for non-disabled people. We believe that this difference can be attributed to the Chinese government's targeted safeguards for the disabled community during the pandemic, including support for disability empowerment, the establishment of a remote interactive decision-making system, fair vaccine distribution and economic protection for PWD. The professionalism of decision-makers and the maintenance of channels of interaction with PWD are also important. All of these changes have benefitted China's public health policy and legal framework. This system, which has six components (governance, prevention, response, knowledge, coordination, and people), enables the country to quickly formulate a series of safeguards for PWD that have a sufficient legal basis. We believe that China's rapidly improving public health policy and legal framework will make a hugely significant impact by alleviating the impact of the COVID-19 pandemic on the PWD community. Countries should pay more attention to discovering the special needs and obstacles of PWD in the COIVD-19 pandemic: in referring to China's experience, they should explore the protection framework for persons with disabilities and adjust it to their own needs on the basis of national conditions.

## Introduction

As of July 9, 2021, the COVID-19 pandemic has caused 185,291,530 infections in more than 200 countries and regions, and 4,010,834 deaths (1). It has become the most severe post-WW2 public health crisis (2).

PWD account for about 15% of the world's total population (3) and are a key population in global public health (4); when compared with non-disabled people, disability makes this group more physically vulnerable and more susceptible to infection, and this is confirmed by a PWD report that shows they are four times more likely to experience poor health (5); in addition, it is also much more difficult for them to be included in health promotion programs (6). In medical care services (especially preventive medical services), there are also general differences between them and the able-bodied (7). These disadvantages mean they face a greater risk of death, even in non-pandemic environments. For example, evidence from the US suggests that adults with any disability are more likely to die than those without disabilities (8).

The COVID-19 pandemic has magnified the special vulnerability of PWD. But PWD face various inequality-related challenges in the previous worldwide COVID-19 emergency response. First of all, a considerable amount of public health information is not accessible, and this may place PWD at a clear disadvantage when they seek to obtain public health information (9). Second, the social distancing measures adopted by some countries in response to COVID-19 may make it difficult for PWD to obtain necessary care services, and this may obstruct their recovery and even endanger their lives (10). Finally, PWD experience considerable disadvantage in the allocation of medical resources in many countries, and the COVID-19 pandemic may further exacerbate this problem (11). The survey results of the global COVID-19 disability rights monitor show that COVID-19 has had a catastrophic impact on PWD worldwide (12). For example, in New York State, data made available on May 28, 2020 shows that people with intellectual disabilities are more than four times more likely to be infected with COVID-19 than non-disabled counterparts (13). In the UK, PWD were 2-3 times more likely to die from COVID-19 in the period January–November 2020 (14).

In addition, the pandemic has had a comprehensive impact on PWD. Their significantly disadvantaged position, which is confirmed by their relative economic status and education levels, has further increased their specific vulnerability in the pandemic (15). This means they are more prone to unemployment, poverty or food insecurity during the pandemic. Both education and work have increasingly shifted online during the pandemic, and this has further widened the digital divide between PWD and non-disabled people (16, 17). Many remote software platforms are not accessible to PWD, and this has further hindered their access and use (18, 19).

The pandemic has now lasted for more than a year. The difficult situation of PWD in the pandemic has increasingly preoccupied decision-makers in various countries. For example, the state of Alabama in the United States quickly canceled ventilator rationing guidelines that were criticized as an example of disability discrimination (20). The Spanish government began to issue disability benefits to PWD affected by COVID-19 (21), and its UK counterpart has prioritized PWD in its vaccine distribution. We calculate that the vaccination rate of PWD in England is 91.2%, which is 1.6% higher than the rate for non-disabled people (22). These actions will help to alleviate the impact of the pandemic on disabled groups.

**Figure 1:**
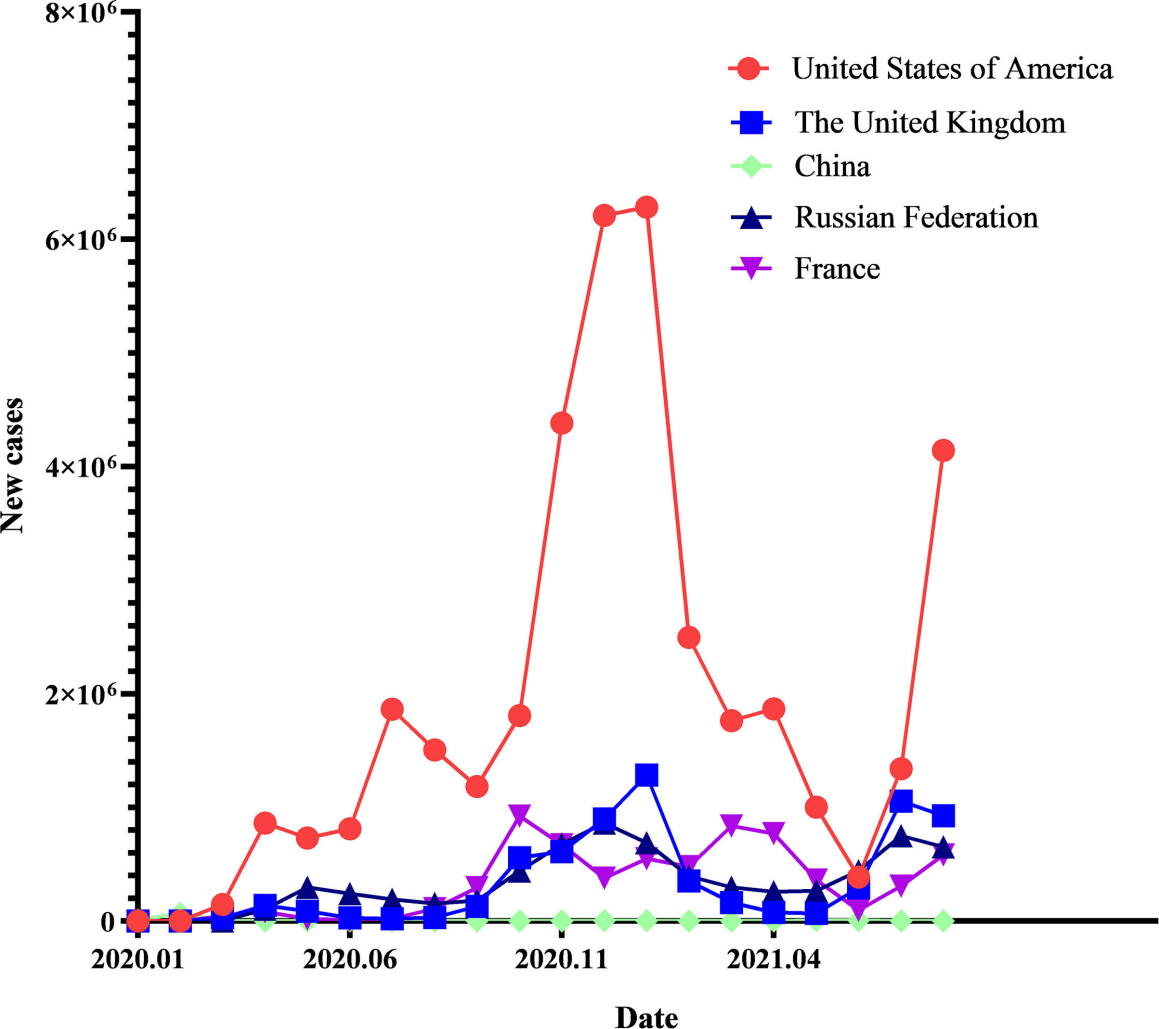
Daily new cases in Britain, China, France, Russia, and the US.

China's effective epidemic prevention measures also deserve attention. As shown in Figure 1, data from the World Health Organization (WHO) shows that China had successfully controlled the spread of the virus in its territory before the first vaccinations were given on March 15, 2021. Data for daily new cases that covers the past through months, also suggests that the spread of the Delta strain, following on from an outbreak in Nanjing, China in July 2021, has been rapidly brought under control.

Information provided by the China Disabled Persons' Federation, the largest disabled persons' rights organization in China, suggests that the infection rate of Chinese PWD may be significantly lower than for non-disabled counterparts (23). After this was announced, many of the country's regions disclosed their own infection data about PWD to the media. This suggests the infection rate of PWD in many regions of China is generally very low, and in some regions (such as Hainan Province which has a population of more than 10 million) has even reached 0 (24).

This article now provides a discussion of China's public health policy and legal framework, and seeks to identify how the government and other policy actors have sought to protect the health of PWD during the COVID-19 pandemic.

## China's Public Health Policy and Legislative Framework

**Figure 2:**
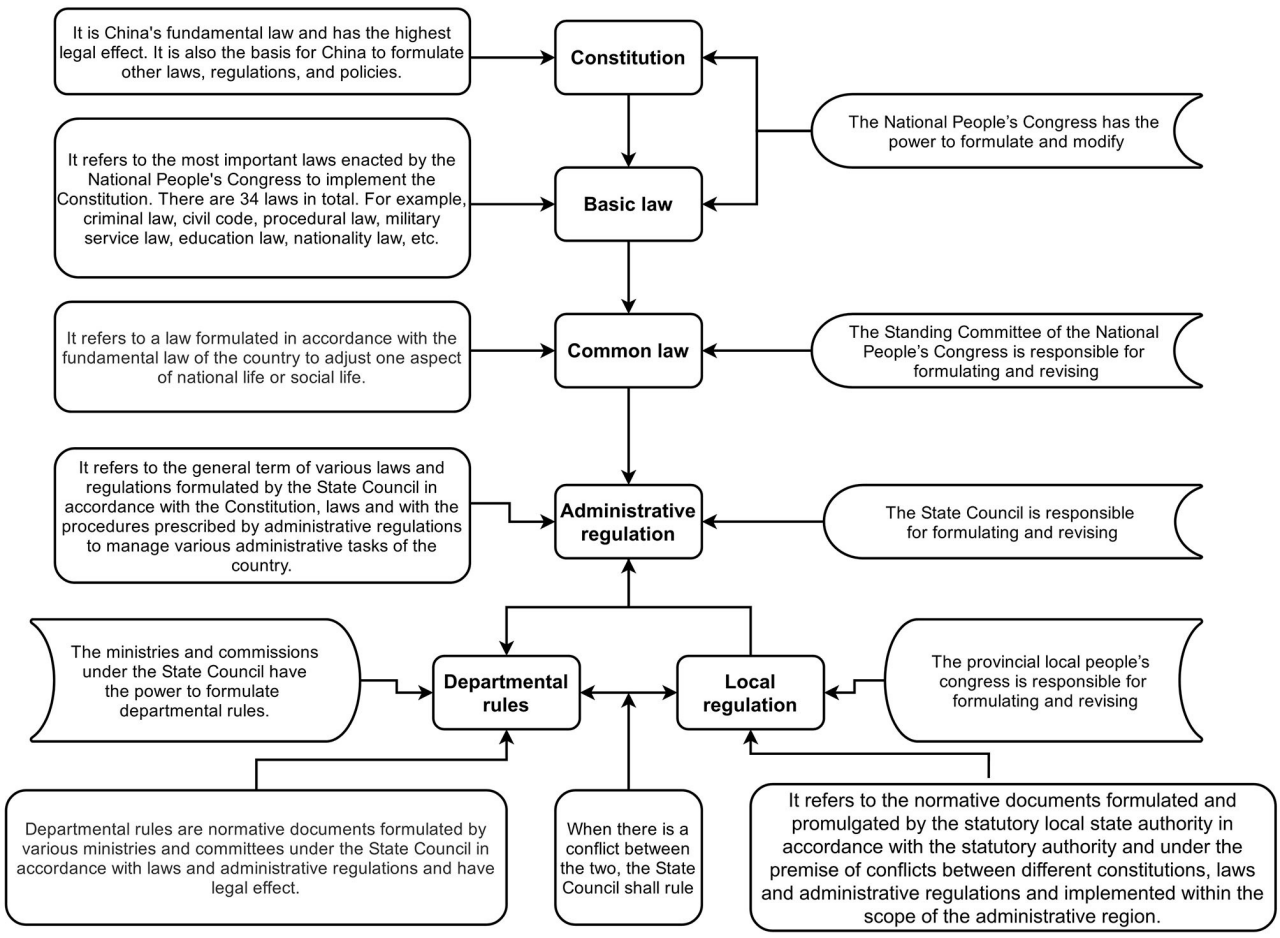
China's legal system.

China is a typical code law country (25). As shown in Figure 2, the constitution is at the top of its legal pyramid and has the highest legal effect. All other laws are formulated and amended in accordance with the Constitution. Below it, there are a series of basic laws, ordinary laws, administrative regulations, departmental rules and local regulations that are positioned in descending order Lower-level law cannot violate the provisions of the upper-level law, and nor can it stipulate the contents of upper-level law (26).

**Figure 3:**
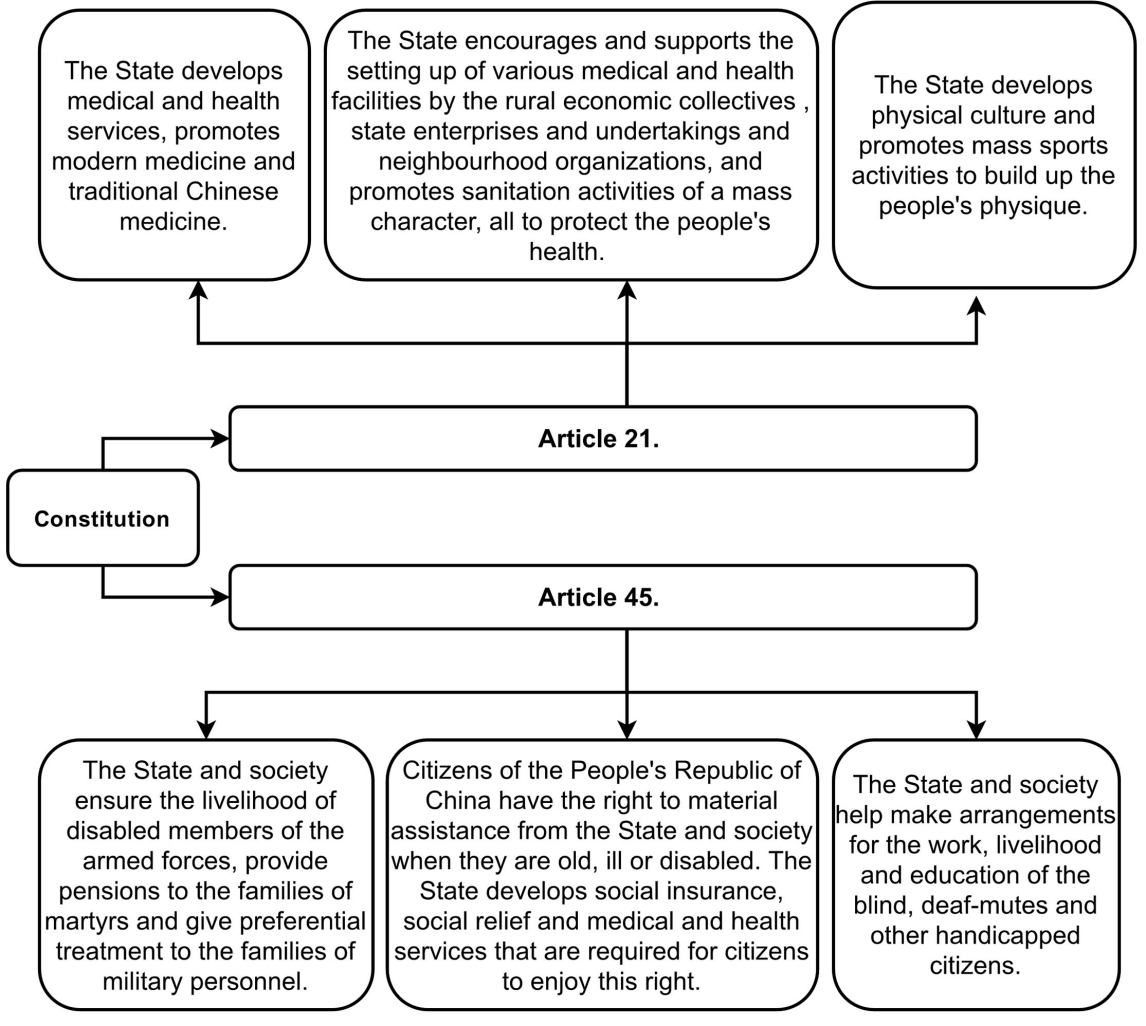
Articles 21 and 45 of the Chinese Constitution.

The Constitution stipulates China's public health undertakings in seven articles. Of these, Articles 21 and 45 define the basic principles of China's health policy and legislation, as shown in Figure 3.

These Articles emphasize that the Government has an obligation to protect people's health, and also requires that the country's health policies and legislation must adhere to the following basic principles: First, to inherit Chinese traditional medicine. The Constitution stipulates that Chinese traditional and modern (Western) medicine have the same important developmental value. Second, the state should apply a series of measures to develop medical and health commitments, including protecting people's health and enhancing their physique. This includes encouraging and supporting actors other than the Government to establish more medical and health facilities, and promoting mass sanitation activities. Third, Chinese citizens have a right to receive help from the state and society when they are old, ill or disabled, and it is established that the State should actively and effectively respond to requests made by such groups, and PWD and disabled soldiers in particular, in a variety of ways.

In applying these principles, the Government has established a huge public health policy and legislative framework. On the basis of referring to the Commonwealth public healthy policy framework (27), we believe that China's public health law and policy framework can be summarized as a system composed of six components, specifically “governance, prevention, response, knowledge, coordination, and people.”

### Governance

An institutional framework for regulating the functions, roles and responsibilities of medical and public health institutions. It includes the management system of doctors and nurses and the management and planning of medical care.

### Prevention

The institutional framework for preventing public health risks. It includes the vaccine management, health norms and protection standards and public health risk reporting systems.

### Response

The emergency system framework used to respond to public health emergencies or infectious disease pandemics. It includes the travel restriction, lockdown and material guarantee systems.

### Knowledge

The institutional framework of public health knowledge. It includes medical and pharmaceutical research norms; drug and medical device research; development and production norms; standard treatment plan research; and promotion systems.

### Coordination

The cooperation and communication system that works with the public health institutions of other countries and/or international health organizations.

### People

The framework of a targeted security system that benefits specific groups. It includes the security system of medical care for disabled people and the relief system for the needy during public health emergencies or infectious disease pandemics.

**Figure 4:**
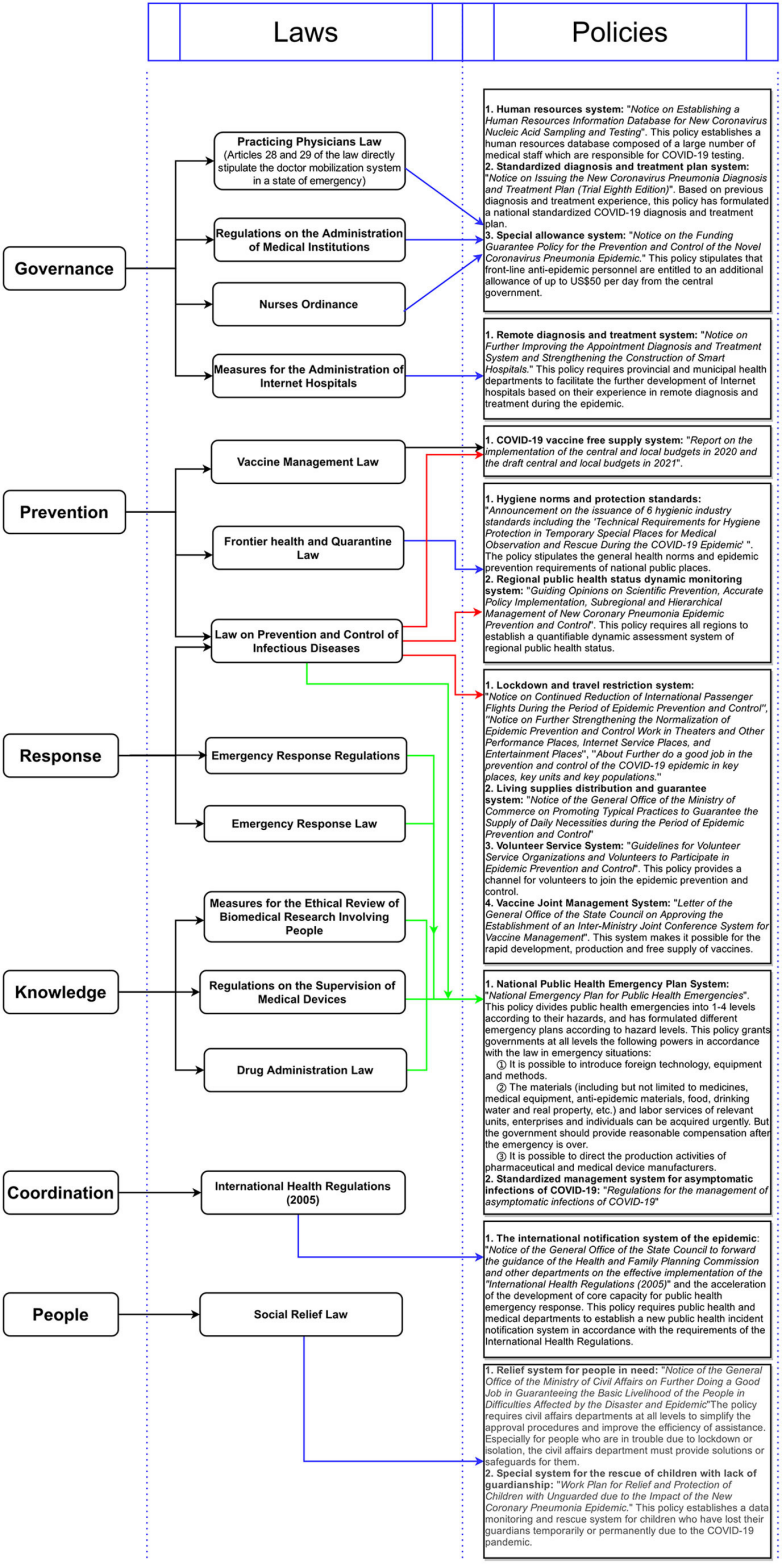
China's public health policy and legislative framework.

In Figure 4, we summarize important laws and policies that are part of the six components, with the aim of making it possible to visualize this framework.

China prepared a complete public health legislative framework before the COVID-19 pandemic that was based on its previous experience of fighting SARS (28). In the PHE period, key elements such as community participation, human resources, material rationing, social blockade and social relief were all prepared at the legislative level. During the pandemic, the Government was able to quickly formulate various safeguard measures for PWD without having to commit substantial amounts of time to revising the law.

## Safeguard Measures for Chinese PWD During the COVID-19 Pandemic

In working within the existing institutional framework, the Government has mainly formulated safeguard measures for PWD by focusing on four aspects, specifically; support for the employment of PWD; remote interactive decision making; the establishment of a fair vaccine system; and economic protection.

### Support the Empowerment of People With Disabilities

The disability group includes multiple sub-groups such as the blind, deaf and intellectually disabled. They have a variety of unique lifestyles and community cultures that are based on their disability characteristics, which makes it difficult for PWD and non-disabled people to understand each other (29). The majority of decision-making bodies are usually made up of non-disabled persons, and this gap between groups is therefore likely to result in the perspectives of disabled people being insufficiently incorporated into relevant systems and legislation. In public health emergencies (PHE), this “blind spot” at the institutional level can be fatal for PWD (9). When the existing system struggles to adjust quickly in PHE, the empowerment of the disabled becomes necessary.

Empowerment is a process that enables people, in their own lives, their communities and their society, to actively address issues they view as important (30). Disability empowerment seeks to enhance the strength of PWD in key elements such as politics, psychology and physiology, and it achieves this by optimizing systems and education and promoting employment in a way that helps to sustain equal participation in social activities and management. This means that empowered PWD have a stronger ability to organize, manage and protect themselves during PHE. At a time when pandemic prevention consumes a substantial amount of the government's administrative and human resources, the independence produced by disability empowerment gains an added importance and significance.

Like most advanced countries, China has always been devoted to disability empowerment, and has therefore sought to provide support and convenience that enables PWD to be employed, become educated and socially participate to the greatest possible extent (31). But China's most important innovation in this area is its “full-time committee system of persons with disabilities,” which requires that every block, community or village must select a full-time committee member of PWD from its disabled residents, and only PWD have the right to vote. People with severe intellectual disabilities or other severely disabled persons can be selected by their immediate family members, who put them forward as candidates. The salaries and social insurance funds of the full-time PWD members are provided by the state.

The main responsibilities of the full-time committee members include (32):

① Investigating the conditions that PWD live in and the challenges they face in their respective jurisdictions; and summarizing their appeals and submitting them to the State authorities.

② Organizing PWD in their respective jurisdictions so that they can participate in recreational and sports activities.

③ Liaising with volunteer organizations, non-profit organizations and relevant authorities to support PWD.

④ Supervising the implementation of preferential policies for PWD and helping the Government to distribute materials to PWD.

⑤ Establishing a network that includes all PWD in their respective jurisdictions.

The China Disabled Persons' Federation initially explored and established this system with the support of the central government in 2014. The original intention of the system was to help unify PWD in each community into a mutual help group; this, it was anticipated, would enhance their power and enable them to live with more dignity (33). The full-time PWD members have therefore actually encouraged disabled persons in their jurisdictions to form small autonomous bodies, where they establish contact networks and organize group activities, and this in turn enables them to collectively express their demands and effectively enhance their autonomy and independence. The autonomous nature of the Commission meant that, although the Government provided funds for full-time members, no Government officials or non-disabled persons actually joined.

In an unexpected development, the empowered disability group did not only enable members to protect themselves, but also enabled them to provide support to others. For example, the blind Mr. Xu, a volunteer, led the dispatch of more than $1 million in emergency supplies during the most difficult period in Wuhan, and also contacted helicopters who conducted 13 “sorties” that delivered supplies to the city (34). After the disabled community showed its power, the Government began to welcome more PWD, including full-time members, to participate in epidemic prevention on a voluntary basis (35).

### Establish a Remote Interactive Decision-Making System

In the field of administrative decision-making, interactive decision making (IDM) refers to the authorities' use of seminars, group discussions and many other techniques to find innovative and supportive solutions to existing problems (36). Studies show that, when addressing risky choices, groups make fewer decision-making mistakes than individuals (37), and are more likely to make faster and better decisions in uncertain environments (38). IDM has therefore become a common practice in administrative decision-making and it is also applied across all levels of government (39). But the urgency of public health emergencies (PHE) usually requires the authorities to make emergency decisions quickly, so as to avoid a rapid escalation of the crisis. For example, in the COVID-19 pandemic, after a team of medical experts determined that COVID-19 could be transmitted from person to person, China's central government imposed a lockdown on the Wuhan, the outbreak epicenter, and its population of 12.33 million was therefore confined indoors in just 72 h. The rapidity of this process made it difficult for the authorities to fully interact with communities in the decision-making stage, and the social isolation caused by epidemic prevention further limited the realization of IDM. PWD and the non-disabled struggle to understand each other in the best of circumstances, and emergency decision-making is likely to further exacerbate mutual miscommunication and misunderstanding.

Chinese PWD mainly participate in decision-making by being invited to key meetings that take place every day. During the COVID-19 pandemic, this mode of became both risky and inefficient. The diversity of the disabled community also meant that many problems that arose during the pandemic were difficult to predict in advance. Remote Interactive decision-making system (RIDM) is an interactive decision-making participation model that the Government applies in an emergency: When PWD realize that there are problems with the relevant decision-making, or require the authorities to make decisions that resolve difficulties faced by PWD, they can call the hotline and submit a request. This is a “decision starter.” The hotline will then transfer the appeals of PWD to the agency that has the authority to make relevant decisions. Officials will then speak to PWD on the phone and will try to quickly develop a solution. Here PWD are “decision-making participants.” In order to prevent relevant needs from being overlooked and/or put to one side, the authorities will ask each “decision starter” if they had received an effective response, and the feedback will then be used in departmental evaluations.

The difference between RIDM and complaint calls is that the latter usually only address the complainant's individual needs; however, the decision made by RIDM can solve all similar problems in relevant jurisdictions.

This RIDM system, which even continuing functioned during January 23–April 8, 2020, the most difficult lockdown period in Wuhan, enabled the authorities to effectively respond to urgent needs. When Wuhan Hospital was overwhelmed by the COVID-19 pandemic, a mutual group of disabled people decided to work with the authorities to establish a temporary supply chain of immunologic agents through the RIDM system. This enabled 500 members of the mutual aid group to obtain immune preparations that the hospital had an exclusive right to supply through a specific pharmacy. As a result, they survived (24). Other patients in Wuhan who need immune preparations can also benefit from this policy.

### Fair Distribution of Vaccines

The generally disadvantaged position of PWD in society means that they require a more equitable vaccine distribution framework to be put in place. Evidence from the United States and Australia shows that disability and the degree of disability are just two factors that affect the likelihood of citizens getting the flu vaccine, and PWD get fair access to vaccination (40, 41). Other related factors include the prejudice of medical staff and travel barriers caused by disability (42, 43), and separate factors include a shortage of vaccines or socioeconomic barriers (44, 45). Although in other countries, such as the US (46), it is acknowledged that the COVID-19 vaccine should be allocated preferentially to disadvantaged groups such as PWD. However, in the case of the US, this has not been achieved.

The overall vaccination rates for PWD in the United States is 4% lower than for non-disabled people, and this gap further widens to 5.3% when the age group 65 years or older is considered (47).

The Chinese government sought to achieve fairness in vaccine distribution in a number of ways. First, by working to solve the problem of vaccine shortage. China was one of the first countries in the world to successfully develop a COVID-19 vaccine (48), and it has rapidly increased its production capacity. On July 16, 2021, the Ministry of Industry and Information Technology of China announced that China's annual output of COVID-19 vaccine has reached 5 billion doses, it would basically ensure the full supply of vaccines for all of China's population (49). In the period mid-March-July, 2021, China vaccinated more than 2 billion people and exported more than 700 million doses of COVID-19 vaccine and stock solution (50). This has meant that China's public health authorities have not even needed to raise the issue of whether to prioritize the distribution of vaccines to disadvantaged groups such as PWD.

Second, the Chinese government has decided to provide free COVID-19 vaccination services to all residents of mainland China (including foreign residents) (51), meaning that vulnerable groups, including PWD, do not have to pay about $30 (USD) for a vaccine dose (52), and this means their economic worries are removed.

The sufficient supply of vaccines means that PWD can decide when to go to the nearest community epidemic prevention station and receive vaccination. However, some community epidemic prevention stations have not completed barrier free transformation, and this is a major barrier for PWD with travel obstacles. China therefore also sought to eliminate the travel barriers of PWD and the possibility of discrimination as a result of cumbersome medical procedures by quickly converting a number of large buses into vaccination vehicles with barrier-free facilities that then directly provided vaccination services to PWD in their own communities (53).

Door-to-door vaccination services are also an effective community response to PWD with serious travel obstacles (54).

But it must be remembered that are still some people in China (Including some PWD) who are unwilling to receive the COVID-19 vaccine. Previous studies suggest this may be due to complacency or concerns about the safety of vaccines (55). At present, China still adheres to the policy of voluntary vaccination, meaning that the authorities have to rely on persuasion and the force of scientific evidence (56). The Government has also distributed some small gifts, such as a bucket of edible oil or a bag of flour, to those who have been vaccinated at epidemic prevention stations (57).

## Provide Reasonable Financial Protection to People With Disabilities in the Pandemic

In the COVID-19 pandemic, long-term and strict social blockade may result in some low-income people completely losing their financial savings. When this happens, they are more willing to break the blockade and go out to find jobs and food (58). Members of the disability group often have low incomes (59), meaning they will be more economically impacted by the pandemic. For example, in China, the blind most often makes a living by providing massage services. But the blind masseur cannot avoid long-term close contact with customers, meaning that he/she was forced to suspend their work during the social blockade. Rent and wages still have to be paid, and this has pushed many blind massage shops closer to bankruptcy.

The Chinese government responded by developing an economic security plan that would assist this particular disadvantaged group. In Hainan Province, for example, the Taxation Bureau reduced or exempted the taxes paid by blind massage shops and blind employees, and also allowed them to defer tax payment. Second, the shops that rented state-owned properties (many properties in Chinese cities belong to the state) were not required to pay 3 months of rent during the social lockdown. Finally, in responding to the cash flow difficulties, the Hainan Disabled Persons' Federation obtained an unconditional economic subsidy of nearly 1,000,000 Yuan (~$150,000) from the provincial government that was then distributed to 114 blind massage shops in the province (60). In responding to the economic difficulties experienced by other persons with disabilities, such as the deaf and mentally impaired, the Chinese government and disability rights organizations jointly formulated a similar economic security plan. The central government addressed the needs of extremely poor disabled persons who have no source of income at all by stipulating that civil affairs departments across all levels are responsible for ensuring that they sustain a basic standard of living during the epidemic prevention period (61).

## Discussion

This article has shown that the professionalism of decision-makers is extremely important for dealing with PHE. In China's unique political system, the heads of governments and functional departments at all levels are industry experts with knowledge of specific industries rather than career politicians (62). This helps to ensure that China's epidemic prevention policies and legislation are, in most cases, prompt, professional, and effective (63). For example, in Hainan Province, where none of the 500,000 disabled residents was infected with COVID-19, the top leader, SHEN Xiaoming, is not only a well-known medical expert in China (64), but is also an Honorary Fellow of American Academy of Pediatrics and Foreign Associate of the U.S-based Institute of Medicine of National Academies (65). His professional background was an invaluable resource that the local authorities were able to draw on as they sought to make correct judgments on various types of PHE, and to optimize the province's public health policies and local legislation in a targeted manner. This is very important for PWD who are less able to resist risks.

Second, China's experience also shows that interaction in the decision-making process is important for PWD. This group has low education levels and economic status (26), and this limits their ability to participate in politics (66), meaning they do not generally exert great influence over decision-making in various countries, including American (67), the United Kingdom (68), and Norway (69). This means the needs of PWD can hardly be included in the emergency response of countries in the early stages of the pandemic. But in reflecting on their own failure to contain the pandemic, affected countries should refer to China's experience and consider how to maintain interaction with the disabled community during the public health policy formulation and legislation process.

Third, we should also pay attention to the positive effects of high social capital on PWD-focused responses to the COVID-19 pandemic. Social capital is an instantiated informal norm that can promote cooperation between individuals (70). In a pandemic, it determines if comply with laws and regulations (71). Research shows that social distancing measures are more decisive and more efficient in areas that have higher levels of social capital (72). People will also work together to comply with government regulations and guidelines if they are self-motivated. In the case of PWD, the experiences of the Wuhan mutual aid group for PWD and the full-time Committee of PWD proves that high levels enable disabled communities to maintain their close ties with each other and the wider society during the COVID-19 pandemic. In addition to significantly improving the self-protection ability of disabled communities during the COVID-19 pandemic, this reduces the risk of PWD being marginalized during the pandemic and provides them with psychological support. However, the discussion should be supplemented by an acknowledgment of studies that suggest that strong family relationships and social gatherings that produce high social capital may become a risk during a pandemic (73). Evidence from Northeastern China shows that most clustered cases in this area occurred in individual families and/or between relatives. This negative effect of social capital should also be acknowledged (74).

In addition, political observers need to consider how to adjust their policies and legislation in order to further prevent PWD from being affected by healthcare discrimination in epidemic prevention. In this article, we find that general shortages of medical resources caused by the pandemic may result in an increased risk that PWD will be discriminated against. Although, we do not ground this claim in evidence collected from the Chinese medical system, it should be noted that Brazil, India, South Korea, Turkey and South Africa have reported that PWD are at high risk of experiencing healthcare discrimination during the pandemic (75–79). Evidence from the American also shows that PWD may be disadvantaged by discriminatory medical rationing when medical resources are scarce (11). In addition, the uneven availability of public health information, which is frequently evidenced in different countries, may also contribute to healthcare discrimination against PWD (80). The above evidence therefore suggests that healthcare discrimination faced by PWD may be a worldwide problem, and all countries need to acknowledge and address this issue.

The fact that China has already begun to raise a question about the fact of this process, that is, whether its example can be imitated or replicated. On the basis of the findings of this article, we reject this proposition, and propose that it should be a point of reference. The foremost consideration in this regard is the unique character of the Chinese political system: unlike the “night watch government” (81) in many countries, which only provides core government functions such as national defense, internal security and prison management, the Chinese government is an engaged “big government,” meaning that it can and is willing to coordinate, exert power and deploy all available domestic resources effectively, and quickly impose necessary restrictions on social activities. Since the early 1980s, various governments, of different stripes and colors, have declared the demise of big government to be “over” (82). However, the political tide will perhaps now turn because both the ongoing pandemic and China's previous experience of fighting swine fever (83) suggest that this form of government has clear comparative institutional advantages in PHE. This feature has, in the view of the authors, been insufficiently acknowledged as a key factor in East Asia's current status as a world leader (84) in the fight against the pandemic. Other factors, including China's abundant human resources and strong industrial production capacity, should also be taken into account, as they have enabled the Chinese government to take prompt measures. We anticipate that when other countries try to replicate China's epidemic prevention experience, they will inevitably encounter shortages of human resources and industrial production capacity, along with severely insufficient government authority.

Finally, we need to acknowledge that the situation of PWD in public health data is still unclear (85). In 2016, some scholars asserted that disability should be counted in public health statistics, and this view grew stronger after the COVID-19 pandemic (86). Our research suggests that too few medical and public health institutions record the disability status of COVID-19 infected persons in statistical data, and we also noted how this has hindered the development of national disability policy and the work of legal researchers during the COVID-19 pandemic (87). We would therefore like to reiterate our request for medical and public health institutions to track disability indicators in statistical data.

## Conclusion

In summary, China has taken a series of effective measures to protect the health of PWD during the COVID-19 pandemic, and its efforts have been considerably aided both by its own national conditions and institutional advantages. Although many of China's specific measures may be difficult to replicate in other countries, we would suggest that other countries should reflect on the measures that the Chinese national government put in place to address the special needs of the disabled group and the specific obstacles that confronted it. Working on the assumption that humans will ultimately have to coexist with COVID-19 (88), we propose that international communities should focus on how to get countries to more closely consider the difficult situation of PWD in PHE, and also explore and construct response plans that are based on their respective national conditions and institutional advantages.

## Data Availability Statement

The original contributions presented in the study are included in the article/supplementary material, further inquiries can be directed to the corresponding author/s.

## Author Contributions

FQ, YW, and QW contributed to conception and design of the study and wrote sections of the manuscript. FQ performed the statistical analysis and wrote the first draft of the manuscript. All authors contributed to manuscript revision, read, and approved the submitted version.

## Funding

This article was an outcome of the Hainan Provincial Philosophy and Social Science 2020 Planning Projects [Grant Number: HNSK(YB) 20-04]. The authors declare that this study received funding from the Hainan Provincial Philosophy and Social Science 2020 Planning Projects. The funder was not involved in the study design, collection, analysis, interpretation of data, the writing of this article or the decision to submit it for publication.

## Conflict of Interest

YW is employed by Danzhou City Construction Investment Co., Ltd. The remaining authors declare that the research was conducted in the absence of any commercial or financial relationships that could be construed as a potential conflict of interest.

## Publisher's Note

All claims expressed in this article are solely those of the authors and do not necessarily represent those of their affiliated organizations, or those of the publisher, the editors and the reviewers. Any product that may be evaluated in this article, or claim that may be made by its manufacturer, is not guaranteed or endorsed by the publisher.

## Abbreviations

PWD: people with disability
PHE: public health emergencies
IDM: interactive decision making.
